# Supplementary material for: Decreasing Incidence of Gastric Cancer with Increasing Time after Helicobacter pylori Treatment: A Nationwide Population-Based Cohort Study
Source: Antibiotics (Basel). 2022 Aug 3;11(8):1052. doi: 10.3390/antibiotics11081052 (PMC9405442; doi:10.3390/antibiotics11081052)
Supplement: Supplementary file 1 [file antibiotics-11-01052-s001.zip › Supple._Tables_and_figure_legends.pdf]

Table S1. Observational Medical Outcomes Partnership Common Data Model drug codes included in the study

Class	Concept IDs
PPI	Rabeprazole (911735); pantoprazole (948078); omeprazole (923645); lansoprazole (929887); esomeprazole (904453); dexlansoprazole (19039926)
Amoxicillin	Amoxicillin (1713332); amoxicillin 500 mg oral capsule (19073187)
Clarithromycin	Clarithromycin (1750500); clarithromycin 500 mg oral tablet (Klaricid) (21113674);
Bismuth	Bismuth subcitrate (958134)
Metronidazole	Metronidazole 250 mg oral tablet (19080187); metronidazole (1707164)
Tetracycline	Tetracycline oral capsule (40087792); tetracycline 250 mg oral capsule (19019852)

Table S2. List of negative control outcomes

Concept ID	Concept Code	Concept Name
378256	46670006	Abnormal reflex
440424	87486003	Aphasia
439237	52684005	Assault
378424	82649003	Astigmatism
261880	46621007	Atelectasis
134118	400190005	Atrophic condition of skin
4224118	40492006	Bladder dysfunction
80509	203465002	Bone cyst
434626	20010003	Borderline personality disorder
438407	78004001	Bulimia nervosa
134765	238108007	Cachexia
4172458	49883006	Candidiasis of skin
436740	17382005	Cervical incompetence
381581	1482004	Chalazion
4307254	423125000	Closed fracture
4047787	123971006	Colles' fracture
198075	240542006	Condyloma acuminatum
73302	64217002	Curvature of spine
4242416	58588007	Cutis laxa
433163	238107002	Deficiency of macronutrients
4047269	229844004	Deformity of foot
133228	80967001	Dental caries
4147672	30415006	Disease due to Papilloma virus

4153380	371160000	Disorder of carotid artery
4140510	3305006	Disorder of lymphatic vessel
433440	78667006	Dysthymia
376132	62909004	Ectropion
440695	302690004	Encopresis
438872	267023007	Excessive eating - polyphagia
78804	27431007	Fibrocystic disease of breast
4131595	12676007	Fracture of radius
74855	33839006	Genital herpes simplex
441788	240532009	Human papilloma virus infection
76737	55434001	Hydrocele
4029582	237793004	Hyperandrogenization syndrome
195212	47270006	Hypercortisolism
438134	77692006	Hypersomnia
140362	36976004	Hypoparathyroidism
4322737	427898007	Infection of tooth
4207688	55184003	Infectious enteritis
79072	266579006	Inflammatory disorder of breast
139099	400097005	Ingrowing nail
4288544	396232000	Inguinal hernia
444191	125593007	Injury of face
444130	125604000	Injury of foot
134222	125597008	Injury of forearm
4029966	128609009	Intracranial aneurysm
437409	127296001	Intracranial injury
4297984	76844004	Local infection of wound

4018050	10443009	Localized infection
439840	1415005	Lymphangitis
4163232	45198002	Mastitis
440389	91138005	Mental retardation
436100	60380001	Narcolepsy
4262178	397732007	Neurogenic dysfunction of the urinary bladder
193874	8009008	Nocturnal enuresis
4171549	419153005	Nodular goiter
442274	52073004	Oligomenorrhea
4215978	414941008	Onychomycosis
4171915	274718005	Orchitis
380731	3135009	Otitis externa
378160	65668001	Otorrhea
192606	60389000	Paraplegia
253796	36118008	Pneumothorax
195501	69878008	Polycystic ovaries
4153877	269406001	Post-traumatic wound infection
434319	44001008	Premature ejaculation
373478	41256004	Presbyopia
199876	73998008	Prolapse of female genital organs
4295888	76641005	Prolapse of intestine
194997	9713002	Prostatitis
4146239	267802000	Pruritus of genital organs
4285569	68633000	Pupillary disorder
81336	57773001	Rectal prolapse
380395	314407005	Retinal dystrophy

141825	267369002	Simple goiter
137054	201066002	Skin striae
434630	3745000	Sleep-wake schedule disorder
4195698	67801009	Tenosynovitis
4339088	87860000	Testicular mass
133141	6020002	Tinea pedis
440814	70070008	Torticollis
435140	67426006	Toxic effect of alcohol
4270490	62994001	Tracheitis
4028970	13617004	Tracheobronchitis
193326	87557004	Urge incontinence of urine
4092565	24976005	Uterine prolapse
140641	57019003	Verruca vulgaris
197036	197811007	Vesicoureteric reflux
261326	75570004	Viral pneumonia

Table S3. Sensitivity analyses of *Helicobacter pylori* treatment and non-treatment cohorts in the general population

Analysis	Lag period	Hazard ratio	95% CI	*P*-value
1:4 PS matching	1 year (main analysis)	0.76	0.50–1.13	0.19
	2 years	0.79	0.51–1.21	0.30
1:1 PS matching	1 year	0.69	0.43–1.08	0.11
	2 years	0.82	0.49–1.36	0.44
PS stratification	1 year	0.78	0.56–1.06	0.12
	2 years	0.79	0.56–1.11	0.17

Abbreviations: PS, propensity score; CI, confidence interval.

Table S4. Sensitivity analyses for individuals from the *Helicobacter pylori* treatment and non-treatment cohorts, aged ≥65 years and men

Analysis	Lag period	Hazard ratio	95% confidence interval	*P*-value
Helicobacter pylori treatment in individuals aged ≥65 years				
1:4 PS matching	1 year (main analysis)	0.87	0.44–1.68	0.69
	2 years	0.56	0.20–1.35	0.23
1:1 PS matching	1 year	0.86	0.39–1.86	0.70
	2 years	0.67	0.22–1.85	0.45
Helicobacter pylori treatment in men				
1:4 PS matching	1 year (main analysis)	0.82	0.51–1.27	0.38
	2 years	0.99	0.60–1.57	0.95
1:1 PS matching	1 year	0.76	0.45–1.27	0.31
	2 years	0.74	0.43–1.27	0.28

Abbreviations: PS, Propensity score

Supplementary figure legends

Figure S1. The overview of cohort construction and study design

Abbreviations: HP, *Helicobacter pylori*

Figure S2. Balance of covariates between target and comparator cohorts before and after propensity score matching

High standardized mean differences (SMDs) in some covariates before propensity score matching were resolved after propensity score matching. Blue dots indicate the SMD of each covariate between the target and comparator cohorts before and after propensity score matching.

Figure S3. A plot of traditional and calibrated significance testing in the analyses

Blue dots indicate the estimates for negative control outcomes. The estimates below the dashed line (gray area) have a $P<0.05$ based on a traditional P-value calculation. The estimates in the orange areas have a $P<0.05$ based on a calibrated P-value calculation.
