# Supplementary figures and images for: Decreasing Incidence of Gastric Cancer with Increasing Time after Helicobacter pylori Treatment: A Nationwide Population-Based Cohort Study
Source: Antibiotics (Basel). 2022 Aug 3;11(8):1052. doi: 10.3390/antibiotics11081052 (PMC9405442; doi:10.3390/antibiotics11081052)

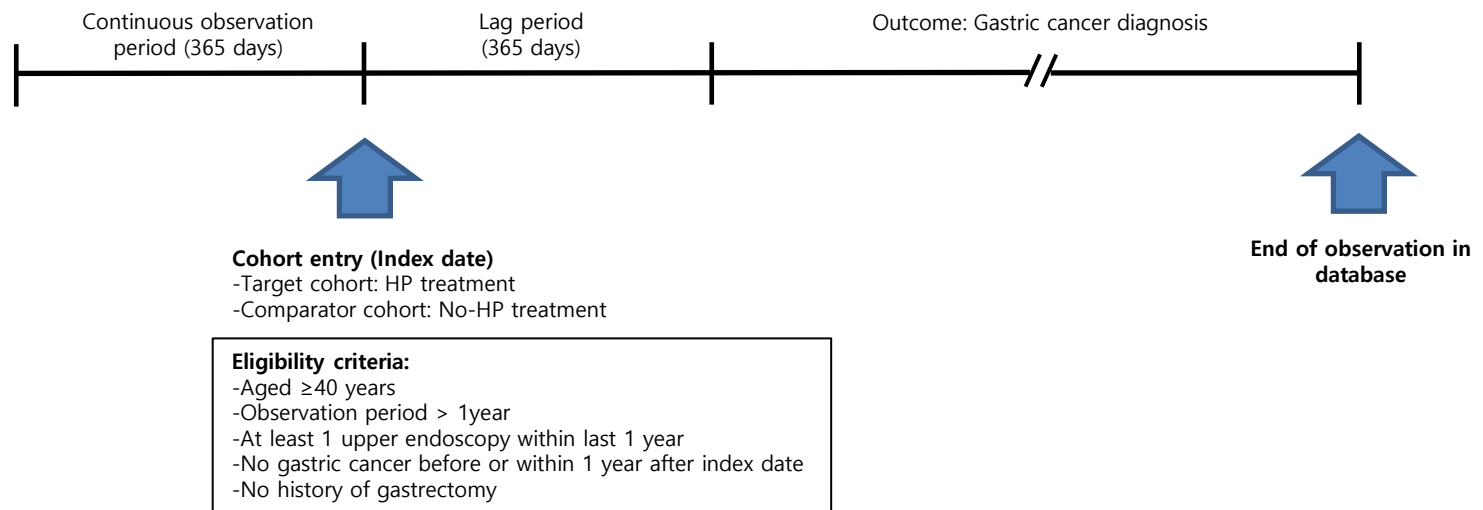

Supplement: Supplementary file 1 [file antibiotics-11-01052-s001.zip › Figure_S1.pdf]

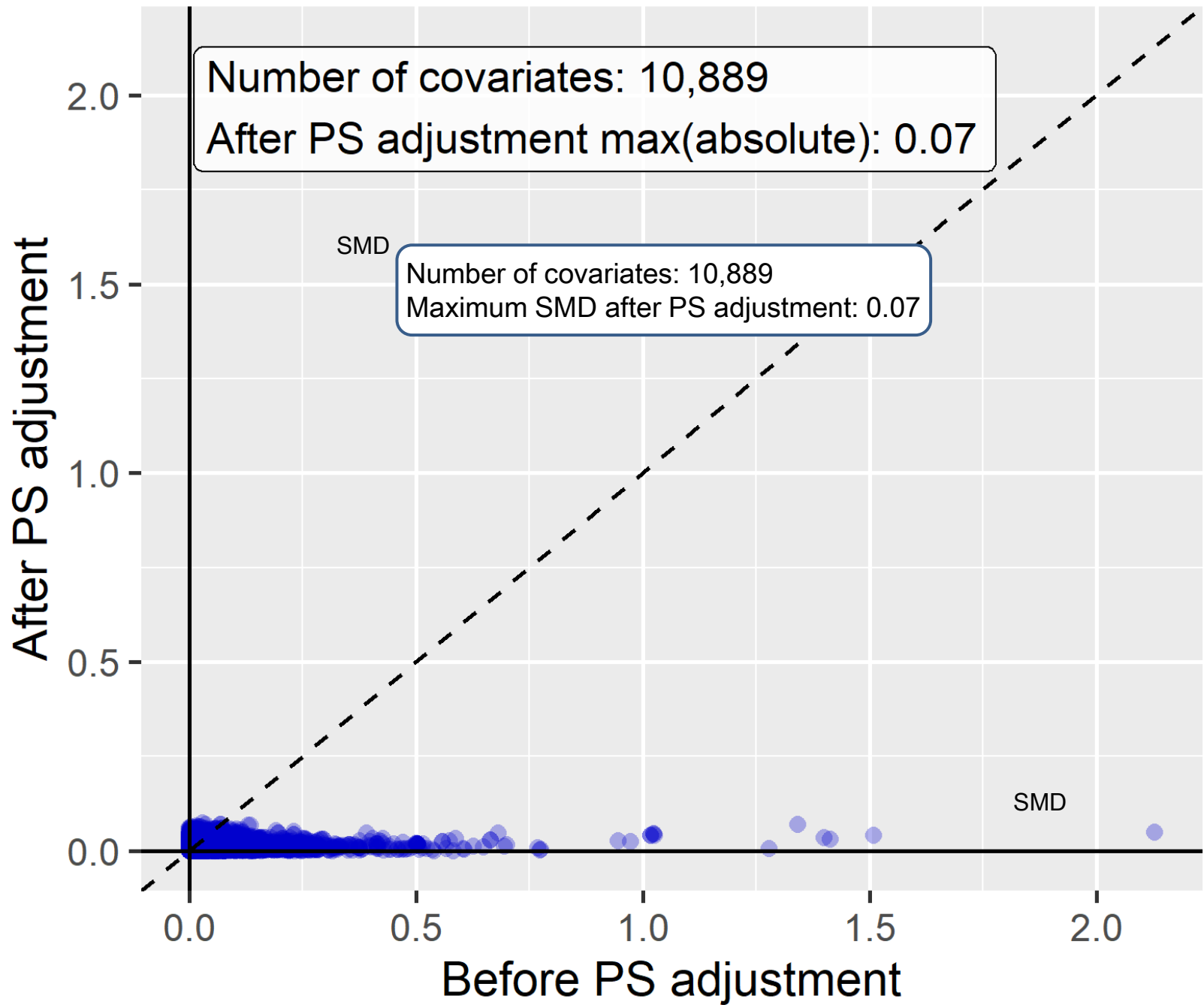

Supplement: Supplementary file 1 [file antibiotics-11-01052-s001.zip › Figure_S2.pdf]

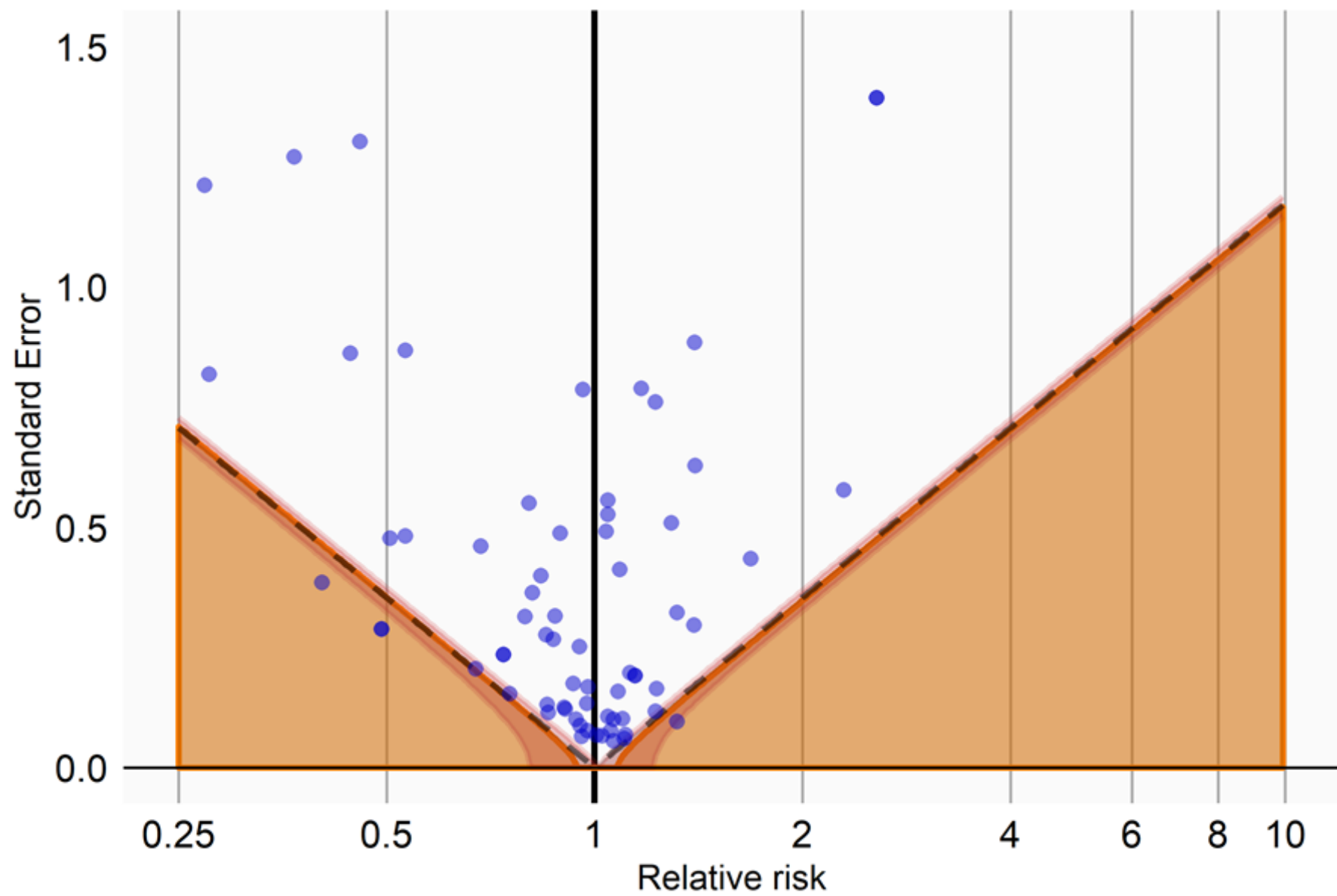

Supplement: Supplementary file 1 [file antibiotics-11-01052-s001.zip › Figure_S3.pdf]
